# Prognostication of prostate cancer based on NUCB2 protein assessment: NUCB2 in prostate cancer

**DOI:** 10.1186/1756-9966-32-77

**Published:** 2013-10-16

**Authors:** Hongtuan Zhang, Can Qi, Andi Wang, Bing Yao, Liang Li, Yuzhuo Wang, Yong Xu

**Affiliations:** 1 National Key Clinical Specialty of Urology, Department of Urology, Second Affiliated Hospital of Tianjin Medical University, Tianjin Key Institute of Urology, 23 Pingjiang Road, Hexi District, Tianjin 300211, China; 2 Department of Radiology, Second Affiliated Hospital of Tianjin Medical University, Tianjin, China; 3 Department of Experimental Therapeutics, British Columbia Cancer Agency, Vancouver, British Columbia, Canada

## Abstract

**Background:**

Nucleobindin 2 (NUCB2) protein, a novel oncoprotein, is overexpressed in breast cancer. To date, there have been no published data regarding the role of NUCB2 protein expression in prostate cancer (PCa). Therefore, this study was performed to investigate the correlations between NUCB2 protein expression and prognosis in patients with PCa.

**Methods:**

Through immunohistochemistry, NUCB2 protein expression was evaluated in 60 benign prostatic hyperplasia (BPH) specimens and 180 PCa specimens. The correlation of NUCB2 protein expression with clinicopathological parameters was assessed using *χ*^2^ analysis. Kaplan-Meier analysis and Cox proportional hazards regression models were used to investigate the correlation between NUCB2 protein expression and prognosis of PCa patients.

**Results:**

The immunohistochemistry results showed that the expression level of NUCB2 in PCa cases was significantly higher than that in BPH tissues (P < 0.001). Moreover, statistical analysis also showed that high NUCB2 protein expression was positively related to seminal vesicle invasion, lymph node metastasis, angiolymphatic invasion, higher Gleason score, biochemical recurrence (BCR), and higher preoperative prostate-specific antigen (PSA). Furthermore, it was also shown that patients with high NUCB2 protein expression had significantly poorer overall survival and BCR- free survival compared with patients with low expression of NUCB2 protein. Multivariate Cox regression analysis revealed that high NUCB2 protein expression level was an independent prognostic factor for overall survival and BCR-free survival of patients with PCa.

**Conclusions:**

NUCB2 protein expression showed a strong association with the potencies of BCR and progression of PCa, and that may be applied as a novel biomarker for the prediction of BCR, and helpful for improving the diagnosis, prognosis and treatment of PCa.

## Background

Prostate cancer (PCa) is the most frequently diagnosed male cancer and the second leading cause of cancer death in men in the United States [1]. Despite the unceasing biomedical research efforts, PCa continues to pose a major public health problem [2]. Serum prostate-specific antigen (PSA), as it is universally known, still remains, in spite of the ongoing criticism, one of the most extensively applied PCa biomarkers [3,4]. Although we have made considerable advances in diagnosis and adjuvant therapy of PCa, many patients develop metastases, the overall survival rate of PCa patients has not been improved markedly. Although some clinical parameters, such as serum PSA levels and Gleason score, may provide some prognostic utility in the treatment settings, there are currently no definitive clinical methods that can reliably predict the responses to clinical therapies for PCa [5-9]. Therefore, it is necessary to identify novel PCa markers to strengthen the efficiency of early diagnosis and to improve the therapeutic strategies of this disease. Evaluation of the expression and role of these proteins in PCa is required for defining molecular and cellular factors associated with PCa aggressiveness and therapy resistance, developing more effective therapeutic interventions, identifying novel PCa biomarkers.

The nucleobindin 2 (NUCB2) gene comprises 14 exons spanning 54,785 nucleotides, with an mRNA of 1,612 nucleotides, of which only nucleotides 246 to 1,508 are translated. The NUCB2 protein contains a 24-amino acid putative signal peptide sequence followed by a 396-amino acid sequence, with very high amino acid sequence homology among rat, mouse, and human species (> 85%) [10]. Structural analyses revealed the presence of several conserved cleavage recognition sites for prohormone convertases within rat NUCB2 sequence, thus suggesting this to be a precursor that gives rise, by differential proteolytic processing, to several active peptides. NUCB2 is proteolytically processed by prohormone to produce at least three peptides, nesfatin-1, nesfatin-2, and nesfatin-3. NUCB2 has a characteristic constitution of functional domains, such as a signal peptide, a Leu/Ile rich region, two Ca^2+^ binding EF-hand domains separated by an acidic amino acid-rich region, and a leucine zipper [11,12], and has a wide variety of basic cellular functions [13-15]. NUCB2 is known to mainly express in key hypothalamic nuclei with proven roles in energy homeostasis [13]. Moreover, recent investigations have indicated that NUCB2 is also expressed in various human peripheral tissues, including the stomach, pancreas, reproductive organs, and adipose tissues, with relevant metabolic functions, suggesting that NUCB2 signaling might participate in adaptative responses and in the control of body functions gated by the state of energy reserves [16].

Protein products of NUCB2 gene have been studied in tumors arising from breast, and stomach [17,18]. To date, no reports investigated the impact of NUCB2 protein expression on the prognosis of patients with PCa. Therefore, the NUCB2 protein expression was measured in PCa tissues and benign prostatic hyperplasia (BPH) tissues by and immunohistochemistry. We studied the correlation between the relative expression of NUCB2 protein and clinicopathological parameters to evaluate its clinical significance. Additionally, we assessed whether NUCB2 protein expression can be used as an independent biomarker for BCR and prognosis of patients with PCa.

## Materials and methods

### Patient and tissue samples

Written informed consent was obtained from all of the patients. The research ethics committee of Tianjin medical university approved the study (TMUhMEC2012015). Formalin-fixed paraffin-embedded samples were obtained from 180 patients with PCa and 60 patients with BPH tissues from patients who were surgically treated in the second hospital of Tianjin medical university, China, between 1999 and 2010. Before radical prostatectomy, none of the PCa patients had received neoadjuvant chemotherapy, androgen deprivation treatment, radiation therapy or immunotherapy. Inclusion criteria were the availability of suitable paraffin blocks for IHC and follow-up information. The histopathology of each specimen was reviewed on the hematoxylin-eosin-stained tissue section to confirm diagnosis. The following biochemical and pathological parameters were recorded: preoperative PSA, Gleason score, PCa stage, lymph node status, angiolymphatic invasion status, surgical margin status, and seminal vesicle invasion status. The TNM staging system was used to describe the extent of PCa in patients (based on the AJCC Cancer Staging Manual, Seventh Edition, 2010, Springer New York, Inc.). The time to biochemical relapse was defined as the period between surgical treatment and the measurement of two successive values of serum PSA level ≥ 0.2 ng/ml. Overall survival was defined as the period from the end of treatment to death or the time of the last follow-up.

### Immunohistochemical staining

NUCB2 immunostaining was performed for all specimens using tissues obtained before treatment. Formalin-fixed, paraffin-embedded tissues were sectioned at 3 μm. The sections were de-waxed in xylene and rehydrated in graded ethanol. Novocastra peroxidase (3% hydrogen peroxide) was used to neutralize endogenous peroxidase activity of the samples for 10 min. NUCB2 staining was carried out by using rabbit polyclonal antibody (Sigma-Aldrich) at a 1:250 dilution, and the samples were incubated for 30 min at 25°C. To reveal the binding of primary antibody by peroxidase staining, the substrate/chromogen, 3,3-diaminobenzidine (DAP), prepared from Novocastra DAP Chromogen and NovaLink DAP Substrate Buffer (Polymer) were used. Finally, the sections were counterstained with hematoxylin, dehydrated, and cleared with xylene, and the sections were mounted for examination. Normal rabbit IgG was used instead of the primary antibody, as a negative control of NUCB2 immunostaining. Human tissue of the breast cancer was used as a positive control for NUCB2 antibody.

### Staining assessment

All of the samples were independently evaluated by two pathologists, who were experienced in evaluating immunohistochemistry and blinded to the clinicopathologic information of these patients. NUCB2 protein expression levels were classified semiquantitatively combining the proportion and intensity of positively stained immunoreactive cells [19]. The percentage of positive-staining tumor cells was scored as follows: 0 (< 5% positive tumor cells), 1 (5-50% positive tumor cells), and 2 (>50% positive tumor cells). Staining intensity was scored as follows: 0 (no staining or only weak staining); 1 (moderate staining); and 2 (strong staining). The sum of the staining intensity score and the percentage score was used to define the NUCB2 protein expression levels: 0-2, low expression and 3-4, high expression. Cases with discrepancies were re-reviewed simultaneously by the original two pathologists and a senior pathologist until a consensus was reached.

### Statistical analysis

The *χ*^2^ test was used to analyze the relationship between the NUCB2 protein expression and the clinicopathological characteristics. Survival curves were plotted using the Kaplan-Meier method and compared using the log-rank test. Survival data were evaluated using univariate and multivariate Cox regression analyses. All statistical analyses were performed using SPSS version 17.0. A p value <0.05 was considered to be statistically significant.

## Results

### NUCB2 protein is overexpressed in PCa tissues

**Figure 1:**
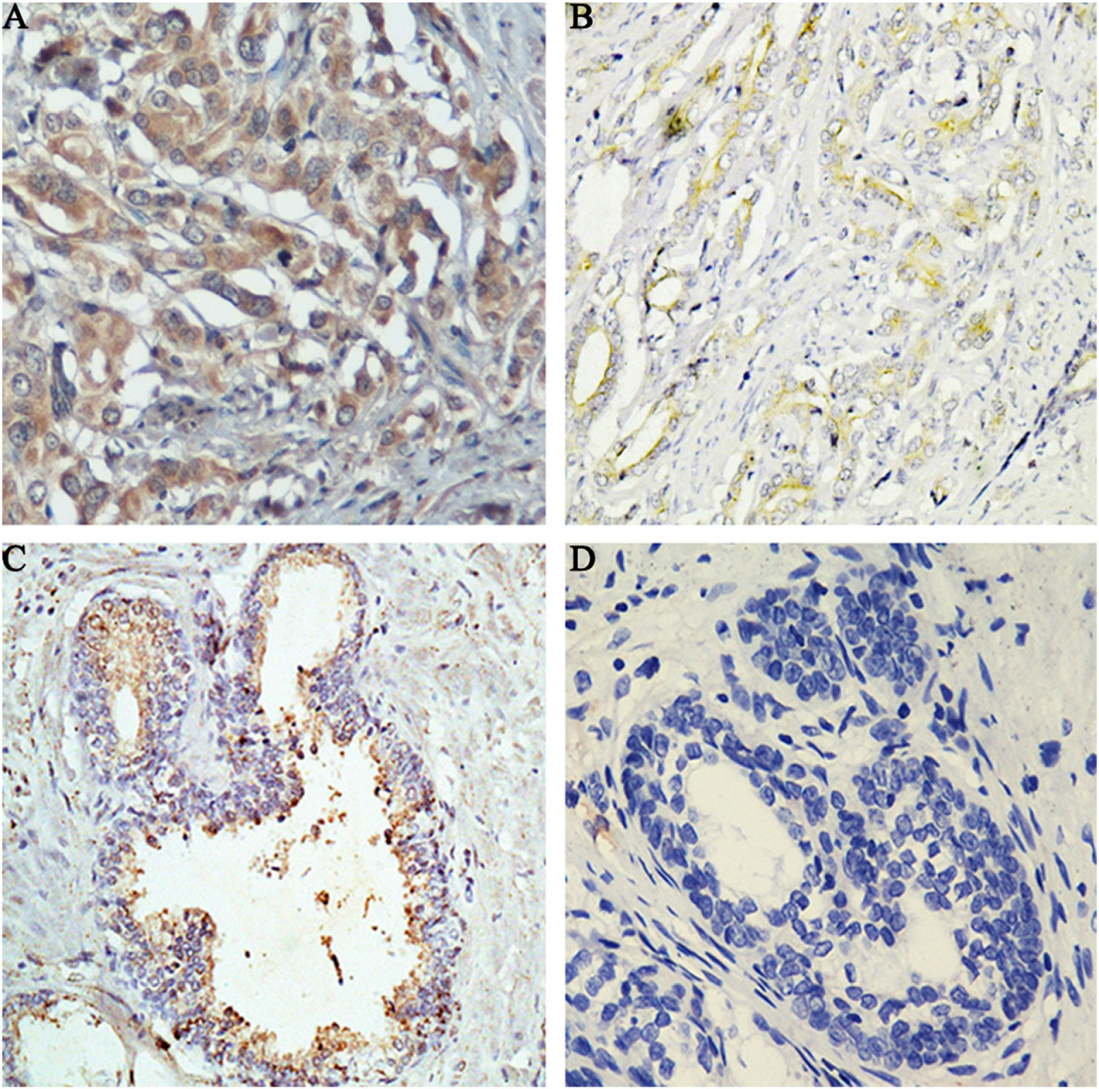
**Immunohistochemical staining for NUCB2 in PCa and benign prostate tissue (original magnification ×200). (A)** High NUCB2 protein expression was found in cytoplasm of PCa tissues. **(B)** Low NUCB2 protein expression was found in cytoplasm of PCa tissues. **(C)** NUCB2 weakly positive staining was found in cytoplasm of benign prostate tissue. **(D)** Negative immunostaining of negative controls with the primary antibody omitted in PCa tissues.

**Figure 2:**
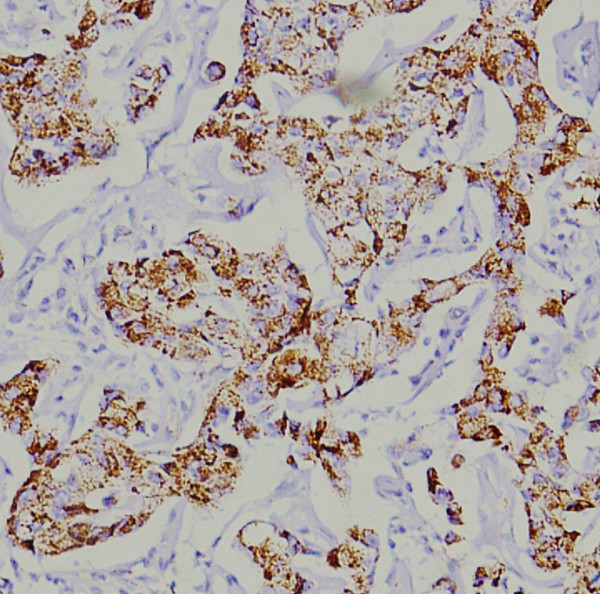
Positive immunostaining of positive controls in breast cancer tissues (original magnification ×200).

A total of 180 PCa patients and 60 BPH patients who were qualified with the inclusion criteria were included in the study. NUCB2 protein expression was high in 4 (6.67%) of 60 patients with BPH and 101 (56.11%) of 180 patients with PCa. NUCB2 protein expression was overexpressed in PCa tissues compared with the BPH tissues, and the difference was statistically significant (P < 0.001) (Table 1). As shown in Figure 1, the NUCB2 staining was localized within the cytoplasm of immunoreactive prostate cells. In the positive control, NUCB2 was mainly positive in the cytoplasm of breast carcinoma cells (Figure 2).

**Table 1:** Expression of NUCB2 protein in prostate specimens

**Groups**	**n**	**NUCB2 protein expression**	**%**	**P**
		**High expression**		
BPH	60	4	6.67%	< 0.001
PCa	180	101	56.11%	

### Association of NUCB2 protein expression with the clinicopathological variables of PCa

We investigated the association between NUCB2 protein expression status and commonly used clinicopathological variables in PCa. The associations between NUCB2 protein expression and clinicopathological variables are shown in Table 2. High expression of NUCB2 protein was significantly associated with seminal vesicle invasion (P = 0.016), the higher level of preoperative PSA (P = 0.006), positive lymph node metastasis (P = 0.022), the positive angiolymphatic invasion (P = 0.042), BCR, and the higher Gleason score (P = 0.017). However, the NUCB2 protein expression was not associated with age, pathological stage, and surgical margin status.

**Table 2:** Clinicopathologic variables and NUCB2 protein expression in 180 PCa patients

**Variable**	**Group**	**NUCB2 protein expression**			**P value**
		**n**	**High**	**Low**	
Age					0.897
	<70	97	54 (55.7%)	43 (44.3%)	
	≥70	83	47 (56.6%)	36 (43.4%)	
Lymph node metastasis					0.022
	Positive	17	14 (82.4%)	3 (17.6%)	
	Negtive	163	87 (53.4%)	76 (46.6%)	
Surgical margin status					0.521
	Positive	14	9 (64.3%)	5 (35.7%)	
	Negtive	166	92 (55.4%)	74 (44.6%)	
Seminal vesicle invasion					0.016
	Positive	35	26 (74.3%)	9 (25.7%)	
	Negtive	145	75 (51.8%)	70 (48.3%)	
PCa stage					0.114
	T1	103	63 (61.2%)	40 (38.8%)	
	T2/T3	77	38 (49.4%)	39 (50.6%)	
Preoperative PSA					0.006
	<4	5	1 (20%)	4 (80%)	
	4-10	64	28 (43.8%)	36 (56.2%)	
	>10	111	72 (64.9%)	39 (35.1%)	
Gleason score					0.017
	<7	99	47 (47.5%)	52 (52.5%)	
	7	34	20 (58.8%)	14 (41.2%)	
	>7	47	34 (72.3%)	13 (27.7%)	
Angiolymphatic invasion					0.042
	Positive	35	25 (71.4%)	10 (28.6%)	
	Negtive	145	76 (52.4%)	69 (47.6%)	
Biochemical recurrence					0.003
	Absence	128	63 (49.2%)	65 (50.8%)	
	Presence	52	38 (73.1%)	14 (26.9%)	

### Correlation of NUCB2 protein expression with BCR-free survival

To investigate the prognostic value of NUCB2 for PCa, we assessed the association between the NUCB2 protein expression and the BCR-free survival duration using a Kaplan–Meier analysis with a log-rank test. The log-rank test showed that the BCR-free survival time of patients with PCa was significantly different between the groups with high NUCB2 protein expression and low NUCB2 protein expression. In patients with PCa, the high NUCB2 protein expression group had a shorter survival duration compared to the low NUCB2 protein expression group. Univariate analysis with Cox proportional hazards model identified 3 prognostic factors: seminal vesicle invasion (P = 0.005), Gleason score (P < 0.001), and NUCB2 protein expression (P < 0.001). The other clinicopathological features, such as age, preoperative PSA, angiolymphatic invasion, lymph node metastasis, surgical margin status, and pathological stage were not statistically significant prognosis factors. By multivariate analysis, we showed that NUCB2 protein expression (P < 0.001), seminal vesicle invasion (P = 0.003), and Gleason score (P < 0.001) were independent prognostic factors for BCR-free survival of PCa patients. The detailed results are present in Table 3.

**Table 3:** Prognostic value of NUCB2 protein expression for the BCR-free survival in univariate and multivariate analyses by Cox regression

**Covariant**	**Univariate analysis**			**Multivariate analysis**		
	**Exp (B)**	**95% CI**	**P value**	**Exp (B)**	**95% CI**	**P value**
NUCB2 protein expression (high/low)	2.306	1.501-3.544	<0.001	2.535	1.643-3.911	<0.001
Gleason score (> 7/7/< 7)	1.703	1.280-2.265	<0.001	1.846	1.384-2.460	<0.001
PSA (>10/4-10/< 4)	1.241	0.705-2.188	0.454			
Age (≥65/< 65)	1.068	0.804-1.419	0.650			
Angiolymphatic invasion (presence/absence)	1.084	0.814-1.443	0.580			
Surgical margin status (presence/absence)	1.017	0.709-1.459	0.925			
PCa Stage (T2, T3/T1)	1.090	0.921-1.291	0.316			
Lymph node metastasis (presence/absence)	1.140	0.850-1.528	0.381			
Seminal vesicle invasion (presence/absence)	1.505	1.132-2.003	0.005	1.538	1.154-2.048	0.003

### Correlation of NUCB2 protein expression with overall survival

To examine the impact of NUCB2 protein overexpression on the overall survival, we first performed univariate analysis of traditional clinicopathologic variables for prognosis. Significant variables in the overall survival analysis included NUCB2 expression (P < 0.001), PCa stage (P < 0.001), Gleason score (P < 0.001), and preoperative PSA (P = 0.001). Multivariate Cox regression analysis enrolling above-mentioned significant parameters showed that NUCB2 protein expression (P < 0.001), PCa stage (P < 0.001), Gleason score (P < 0.001), and preoperative PSA (P < 0.001) were independent prognostic factors for overall survival of patients with PCa. The detailed results are shown in Table 4.

**Table 4:** Prognostic value of NUCB2 protein expression for the overall survival in univariate and multivariate analyses by Cox regression

**Covariant**	**Univariate analysis**			**Multivariate analysis**		
	**Exp (B)**	**95% CI**	**P value**	**Exp (B)**	**95% CI**	**P value**
NUCB2 protein expression (high/low)	2.978	1.516-6.181	<0.001	3.152	1.317-6.214	<0.001
Gleason score (> 7/7/< 7)	2.526	1.788-3.568	<0.001	2.014	1.217-2.869	<0.001
PSA (>10/4-10/< 4)	2.034	1.338-23.092	0.001	1.989	1.292-3.053	<0.001
Age (≥65/< 65)	1.282	0.917-1.792	0.146			
Angiolymphatic invasion (presence/absence)	1.373	0.813-2.319	0.235			
Surgical margin status (presence/absence)	1.101	0.703-1.724	0.674			
PCa Stage (T2, T3/T1)	4.131	2.888-5.911	<0.001	3.671	2.656-5.715	<0.001
Lymph node metastasis (presence/absence)	1.044	0.746-1.462	0.800			
Seminal vesicle invasion (presence/absence)	1.358	0.956-1.928	0.087			

## Discussion

PCa is not a single disease, but an umbrella under which a plethora of heterogeneous diseases is hidden. These range from indolent localized tumors, to aggressive metastatic diseases [20-22]. Metastatic hormone-refractory PCa is the most aggressive form, and is generally associated with very poor prognosis. An accurate and early diagnosis is essential for efficient management of PCa [23-25]. Therefore, to complement improvements in the clinical management, substantial progress in the diagnostic pathway of PCa is urgently needed [26-28]. So evaluation of the expression and role of potential proteins in PCa is required for defining molecular and cellular factors associated with PCa aggressiveness and therapy resistance, developing more effective therapeutic interventions, and identifying novel PCa biomarkers.

Our previous reports indicated that NUCB2 mRNA was upregulated in PCa tissues [29,30]. The data revealed that NUCB2 mRNA may be an independent prognostic factor for BCR-free survival in patients with PCa [29,30]. To date, the associations between NUCB2 protein overexpression and the prognosis of PCa have not been reported. This is the first study to investigate the impact of NUCB2 protein overexpression on the prognosis of PCa based on a relatively large number of clinical samples. In this study, we analyzed NUCB2 protein expression in 180 patients with PCa using immunohistochemistry. We demonstrated, here, that NUCB2 is overexpressed in a large proportion of patients with PCa and high NUCB2 protein expression correlated with the disease progression and poor clinical outcome in PCa. Furthermore, NUCB2 proved to be an independent molecular biomarker of prognosis in PCa and may become a novel molecular target in the strategies for the prognosis of this disease.

We analyzed the association between NUCB2 protein expression and traditional clinicopathogical characteristics in PCa. We observed that the NUCB2 protein levels were significantly higher in PCa tissues compared to those in BPH tissues. We also found that expression of NUCB2 protein expression was significantly associated with seminal vesicle invasion, the higher level of preoperative PSA, positive lymph node metastasis, the positive angiolymphatic invasion, BCR, and the higher Gleason score. These observations support the hypothesis that NUCB2 may function as an oncogene in PCa and that NUCB2 may play an important role in the tumorigenesis of PCa. The data showed that NUCB2 protein overexpression was associated with poor overall and BCR-free survival. These results suggest that high NUCB2 protein expression plays an important role in the progression of PCa and is significantly associated with a poor prognosis independently of other factors. This raises the possibility that NUCB2 may be a prognostic parameter for PCa that is as or more reliable than the clinicopathologic factors currently in use and suggests the possibility to use NUCB2 in individualization of both patient prognosis and therapy.

In the Kaplan–Meier survival analysis, the BCR-free survival period of patients with PCa with high NUCB2 protein expression was significantly shorter than that of patients with low NUCB2 expression. Univariate analyses showed that high NUCB2 protein expression in PCa is significantly associated with the BCR-free survival rate. Moreover, multivariate analysis demonstrated that high NUCB2 protein expression is an independent risk factor in the prognosis of PCa patients. These results suggest that the detection of increased NUCB2 protein expression might help identify PCa patients with a poor prognosis and could, therefore, be a novel prognostic marker for PCa patients. The precise molecular mechanisms behind the altered expression of NUCB2 in PCa are unclear. Additional studies to investigate the real molecular mechanisms of altered expression of NUCB2 in the development or progression of PCa are essential.

Currently, the advantages of serum PSA as a general PCa biomarker are viewed with intense skepticism [31,32]. A variety of algorithms and nomograms that calculate the probabilities of overall and BCR-free survival after treatment have been used in order to direct clinicians into the most suitable treatment options for PCa patients [33]; nonetheless patients still present unforeseen disease course patterns. The present study shows that NUCB2 protein expression can improve PCa management by making available important and independent differential prognostic information. The results indicated that NUCB2 could constitute a molecular prognostic biomarker for PCa patients, identifying who are more likely to have higher risk of BCR and need receive a more aggressive treatment. Our findings could help establish a more personalized medicine-focused approach.

Our study has some limitations. The sample size is not large enough. To solve this problem, a randomized study investigating the association between NUCB2 protein expression and prognosis should be conducted to confirm whether NUCB2 could be used as a novel predictor of overall survival and BCR-free survival. Advanced castration-resistant PCa has not been studied in this study. We will study whether NUCB2 protein expression can provide significant information for the differential discrimination of early localized disease from advanced castration-resistant PCa patients in future. In summary, this is the first study to show an association between NUCB2 protein overexpression and PCa. The results showed that NUCB2 protein overexpression is an independent factor in overall survival and BCR-free survival prognosis and that it may be an important biomarker.

## Conclusions

Taken together, high NUCB2 protein expression in PCa is strongly correlated with seminal vesicle invasion, lymph node metastasis, angiolymphatic invasion, Gleason score, and preoperative PSA. The present results revealed that NUCB2 is an independent prognostic factor for overall survival and BCR-free survival in patients with PCa. Our findings suggest that NUCB2 protein might be used as a new biomarker and a potential therapeutic target for PCa.

## Consent

Written informed consent was obtained from the patient for publication of this report and any accompanying images.

## Abbreviations

NUCB2: Nucleobindin 2; PCa: Prostate cancer; PSA: Prostate-specific antigen; BCR: Biochemical recurrence; DAP: 3, 3-diaminobenzidine; CI: Confidence interval.

## Competing interests

The authors declare that they have no competing interests.

## Authors’ contributions

ZH, QC and XY conceived and designed the study, performed the experiments and wrote the paper. ZH, YB, WY and XY contributed to the writing and to the critical reading of the paper. ZH, QC, LL and WA performed patient collection and clinical data interpretation. ZH, WA, and LL participated performed the statistical analysis. All authors read and approved the final manuscript.

## References

[B1] Siegel R Naishadham D Jemal A Cancer statistics, 2013 CA Cancer J Clin 2013 63 1 11 30 10.3322/caac.21166 23335087

[B2] Jemal A Bray F Center MM Ferlay J Ward E Forman D Global cancer statistics CA Cancer J Clin 2011 61 2 69 90 10.3322/caac.20107 21296855

[B3] Schroder FH Hugosson J Roobol MJ Tammela TL Ciatto S Nelen V Kwiatkowski M Lujan M Lilja H Zappa M Denis LJ Recker F Berenguer A Maattanen L Bangma CH Aus G Villers A Rebillard X van der Kwast T Blijenberg BG Moss SM De Koning HJ Auvinen A Screening and prostate-cancer mortality in a randomized European study N Engl J Med 2009 360 13 1320 1328 10.1056/NEJMoa0810084 19297566

[B4] Stephan C Jung K Lein M Diamandis EP PSA and other tissue kallikreins for prostate cancer detection Eur J Cancer 2007 43 13 1918 1926 10.1016/j.ejca.2007.06.006 17689069

[B5] Eisenberger MA Blumenstein BA Crawford ED Bilateral orchiectomy with or without flutamide for metastatic prostate cancer N Engl J Med 1998 339 15 1036 1042 10.1056/NEJM199810083391504 9761805

[B6] Mengus C Le Magnen C Trella E Yousef K Bubendorf L Provenzano M Bachmann A Heberer M Spagnoli GC Wyler S Elevated levels of circulating IL-7 and IL-15 in patients with early stage prostate cancer J Transl Med 2011 9 162 10.1186/1479-5876-9-162 21943235 PMC3191336

[B7] Berinstein NL Karkada M Morse MA Nemunaitis JJ Chatta G Kaufman H Odunsi K Nigam R Sammatur L MacDonald LD Weir GM Stanford MM Mansour M First-in-man application of a novel therapeutic cancer vaccine formulation with the capacity to induce multi-functional T cell responses in ovarian, breast and prostate cancer patients J Transl Med 2012 10 156 10.1186/1479-5876-10-156 22862954 PMC3479010

[B8] Pinto A Merino M Zamora P Redondo A Castelo B Espinosa E Targeting the endothelin axis in prostate carcinoma Tumor Biol 2012 33 2 421 426 10.1007/s13277-011-0299-6 22203496

[B9] Huo Q Litherland SA Sullivan S Hallquist H Decker DA Rivera-Ramirez I Developing a nanoparticle test for prostate cancer scoring J Transl Med 2012 10 44 10.1186/1479-5876-10-44 22404986 PMC3337274

[B10] Garcia-Galiano D Navarro VM Gaytan F Tena-Sempere M Expanding roles of NUCB2/nesfatin-1 in neuroendocrine regulation J Mol Endocrinol 2010 45 5 281 290 10.1677/JME-10-0059 20682642

[B11] Miura K Titani K Kurosawa Y Kanai Y Molecular cloning of nucleobindin, a novel DNA-binding protein that contains both a signal peptide and a leucine zipper structure Biochem Biophys Res Commun 1992 187 1 375 380 10.1016/S0006-291X(05)81503-7 1520323

[B12] Barnikol-Watanabe S Gross NA Götz H Henkel T Karabinos A Kratzin H Barnikol HU Hilschmann N Human protein NEFA, a novel DNA binding/EF-hand/leucine zipper protein: molecular cloning and sequence analysis of the cDNA, isolation and characterization of the protein Biol Chem Hoppe Seyler 1994 375 8 497 512 10.1515/bchm3.1994.375.8.497 7811391

[B13] Oh-I S Shimizu H Satoh T Okada S Adachi S Inoue K Eguchi H Yamamoto M Imaki T Hashimoto K Tsuchiya T Monden T Horiguchi K Yamada M Mori M Identification of nesfatin-1 as a satiety molecule in the hypothalamus Nature 2006 443 7112 709 712 10.1038/nature05162 17036007

[B14] Taniguchi N Taniura H Niinobe M Takayama C Tominaga-Yoshino K Ogura A Yoshikawa K The postmitotic growth suppressor necdin interacts with a calcium-binding protein (NEFA) in neuronal cytoplasm J Biol Chem 2000 275 41 31674 31681 10915798 10.1074/jbc.M005103200

[B15] Islam A Adamik B Hawari FI Ma G Rouhani FN Zhang J Levine SJ Extracellular TNFR1 release requires the calcium-dependent formation of a nucleobindin 2-ARTS-1 complex J Biol Chem 2006 281 10 6860 6873 10.1074/jbc.M509397200 16407280

[B16] García-Galiano D Navarro VM Gaytan F Tena-Sempere M Expanding roles of NUCB2/nesfatin-1 in neuroendocrine regulation J Mol Endocrinol 2010 45 5 281 290 10.1677/JME-10-0059 20682642

[B17] Kalnina Z Silina K Bruvere R Gabruseva N Stengrevics A Barnikol-Watanabe S Leja M Line A Molecular characterisation and expression analysis of SEREX-defined antigen NUCB2 in gastric epithelium, gastritis and gastric cancer Eur J Histochem 2009 53 1 7 18 19351608 10.4081/ejh.2009.7

[B18] Suzuki S Takagi K Miki Y Onodera Y Akahira J Ebata A Ishida T Watanabe M Sasano H Suzuki T Nucleobindin 2 in human breast carcinoma as a potent prognostic factor Cancer Sci 2012 103 1 136 143 10.1111/j.1349-7006.2011.02119.x 21988594 PMC11164150

[B19] Xiao M Jia S Wang H Wang J Huang Y Li Z Overexpression of LAPTM4B: an independent prognostic marker in breast cancer J Cancer Res Clin Oncol 2013 139 4 661 667 10.1007/s00432-012-1368-y 23292099 PMC11824342

[B20] Bracarda S De Cobelli O Greco C Prayer-Galetti T Valdagni R Gatta G De Braud F Bartsch G Cancer of the prostate Crit Rev Oncol Hematol 2005 56 3 379 396 10.1016/j.critrevonc.2005.03.010 16310371

[B21] Zhou Y Su Z Huang Y Sun T Chen S Wu T Chen G Xie X Li B Du Z The Zfx gene is expressed in human gliomas and is important in the proliferation and apoptosis of the human malignant glioma cell line U251 J Exp Clin Cancer Res 2011 30 114 10.1186/1756-9966-30-114 22185393 PMC3259083

[B22] Li Z Tanaka H Galiano F Glass J Anticancer activity of the iron facilitator LS081 J Exp Clin Cancer Res 2011 30 34 10.1186/1756-9966-30-34 21453502 PMC3077325

[B23] Carroll PR Early stage prostate cancer-do we have a problem with over-detection, overtreatment or both? J Urol 2005 173 4 1061 1062 10.1097/01.ju.0000156838.67623.10 15758699

[B24] Yang L You S Kumar V Zhang C Cao Y In vitro the behaviors of metastasis with suppression of VEGF in human bone metastatic LNCaP-derivative C4-2B prostate cancer cell line J Exp Clin Cancer Res 2012 31 40 10.1186/1756-9966-31-40 22549243 PMC3511813

[B25] Xiang YZ Xiong H Cui ZL Jiang SB Xia QH Zhao Y Li GB Jin XB The association between metabolic syndrome and the risk of prostate cancer, high-grade prostate cancer, advanced prostate cancer, prostate cancer-specific mortality and biochemical recurrence J Exp Clin Cancer Res 2013 32 9 10.1186/1756-9966-32-9 23406686 PMC3598969

[B26] Ribeiro R Monteiro C Cunha V Oliveira MJ Freitas M Fraga A Príncipe P Lobato C Lobo F Morais A Silva V Sanches-Magalhães J Oliveira J Pina F Mota-Pinto A Lopes C Medeiros R Human periprostatic adipose tissue promotes prostate cancer aggressiveness in vitro J Exp Clin Cancer Res 2012 31 32 10.1186/1756-9966-31-32 22469146 PMC3379940

[B27] Filella X Foj L Milà M Augé JM Molina R Jiménez W PCA3 in the detection and management of early prostate cancer Tumor Biol 2013 34 3 1337 1347 10.1007/s13277-013-0739-6 23504524

[B28] Delgado PO Alves BC Gehrke Fde S Kuniyoshi RK Wroclavski ML Del Giglio A Fonseca FL Characterization of cell-free circulating DNA in plasma in patients with prostate cancer Tumor Biol 2013 34 2 983 986 10.1007/s13277-012-0634-6 23269609

[B29] Zhang H Qi C Li L Luo F Xu Y Clinical significance of NUCB2 mRNA expression in prostate cancer J Exp Clin Cancer Res 2013 32 1 56 10.1186/1756-9966-32-56 23958433 PMC3751731

[B30] Zhang H Qi C Wang A Li L Xu Y High expression of nucleobindin 2 mRNA: an independent prognostic factor for overall survival of patients with prostate cancer Tumor Biol 2013 DOI: 10.1007/s13277-013-1268-z 10.1007/s13277-013-1268-z 24092574

[B31] Diamandis EP Prostate cancer screening with prostate-specific antigen testing: more answers or more confusion? Clin Chem 2010 56 3 345 351 10.1373/clinchem.2009.140046 20093554

[B32] Shiraishi T Terada N Zeng Y Suyama T Luo J Trock B Kulkarni P Getzenberg RH Cancer/testis antigens as potential predictors of biochemical recurrence of prostate cancer following radical prostatectomy J Transl Med 2011 9 153 10.1186/1479-5876-9-153 21917134 PMC3184272

[B33] Shariat SF Karakiewicz PI Suardi N Kattan MW Comparison of nomograms with other methods for predicting outcomes in prostate cancer: a critical analysis of the literature Clin Cancer Res 2008 14 14 4400 4407 10.1158/1078-0432.CCR-07-4713 18628454

